# A submandibular gossypiboma mimicking a salivary fistula: a case report

**DOI:** 10.4076/1757-1626-2-6413

**Published:** 2009-07-27

**Authors:** Abu-Ella Amr

**Affiliations:** Department of SurgerySahel Educational Hospital, CairoEgypt

## Abstract

Retained gauze swabs in the neck have seldom been reported. We present the case of a 27-years-old man who suffered a persistent discharging sinus for 8 years following excision of a right submandibular gland. Computed tomography fistulography was done showing a Blind track ending into a cavity just beneath the floor of mouth. Neck exploration eventually revealed 2 gauze swabs that were tightly packed in the area of submandibular duct. This article further emphasizes the importance of sound operative room practice to avoid this serious problem.

## Introduction

Gossypiboma is the technical term for a retained surgical sponge (RSS). The true incidence of RSS is difficult to determine due to underreporting [1,2]. However, it has been reported as 1 in 100 to 3000 for all surgical interventions and 1 in 1000 to 1500 for intraabdominal operations [3]. Retained surgical sponges can lead to significant medical and legal problems between the patient and the doctor. Moreover, a wrong preoperative diagnosis can lead to unnecessary invasive diagnostic procedures and operations [4].

## Case presentation

**Figure 1.**
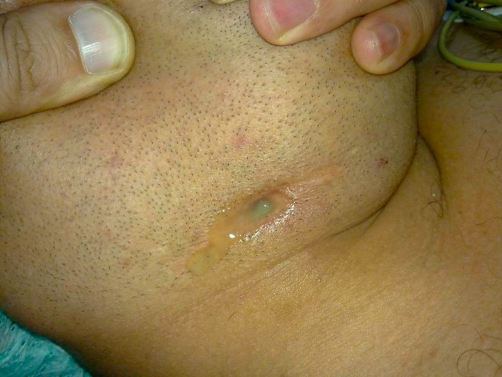
Discharging sinus showing copious discharge.

A 27-year-old Egyptian man working as a clerk presented with a discharging sinus in the right side of his neck (Figure 1). The man had a right submandibular sialadenectomy 8 years before presentation. The patient reported the sinus to appear after removing a drain which has been placed. This surgery was performed elsewhere. He also complained of pain below his chin and an increase in the amount of discharge on mastication. No history of fever. The patient reported receiving several trials of antibiotic therapy in an attempt to cure his condition. He had an X-ray done before presentation. The report of the excised gland pathological examination was not available.

On examination the man was 90 kilograms body weight and 182 centimeters height, there was a healed scar of previous surgery and a discharging sinus in the right submandibular region the discharge was purulent. No palpable swellings or enlarged cervical lymph nodes were found. Bimanual examination was done and there were no palpable swelling in the floor of mouth. A provisional diagnosis of a salivary fistula was considered.

**Figure 2.**
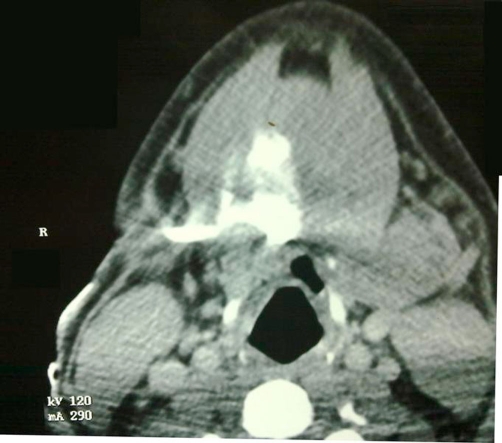
CT fistulogram showing the cavity below floor of mouth.

Neck X-ray showed no abnormality. A CT fistulogram was undertaken (Figure 2), which showed a blind ending track leading to cavity just beneath the right side of floor of mouth. It also confirmed excision of the right submandibular gland.

**Figure 3.**
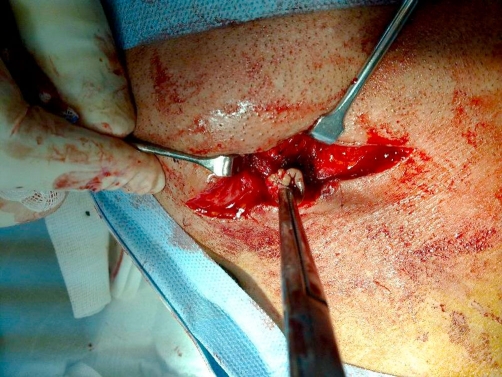
Early dissection of the well formed track.

**Figure 4.**
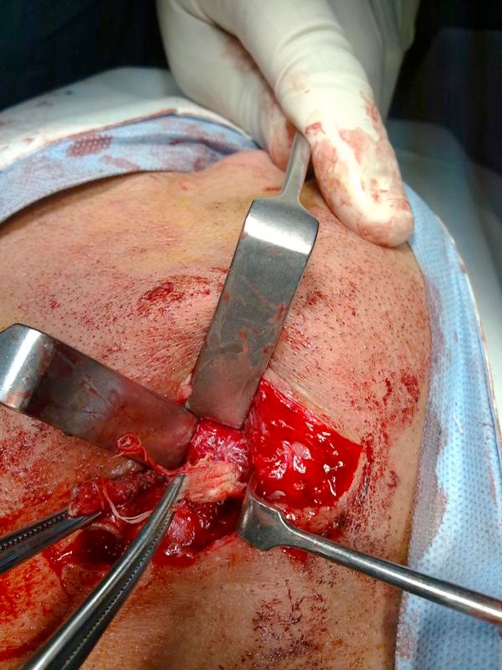
The tightly packed sponges.

**Figure 5.**
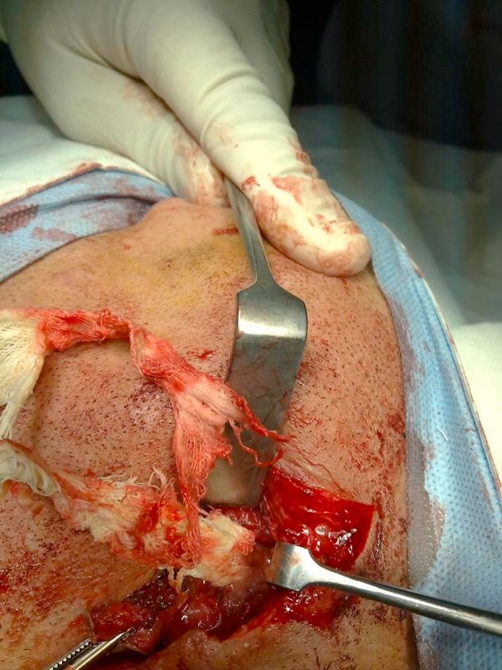
Extraction of the sponge.

A decision to perform neck exploration was undertaken. During surgery gentle probing of the track was done then dissection was carried out revealing the sinus track passing medial the digastric muscle and up to sublingual area (Figure 3). The tracks ended into a cyst below the mandible in the area where we typically ligate and divide the submandibular duct. On opening the cyst a purulent discharge appeared and irrigation of the cavity further revealed white fabric fibers (Figure 4). With traction on these fibers two small sponges were found and removed (Figure 5). The track was then excised and the cavity and wound copiously irrigated with saline. A suction drain was inserted and wound closed. The patient had an uneventful postoperative course and primary healing was achieved.

## Discussion

Retained surgical sponges in the neck are much less reported than intrabdominal ones. Nevertheless they can produce a variety of postoperative complications and can lead to diagnostic misinterpretation e.g. tumor recurrence [5]. Our patient was suspected of having a salivary fistula, another rare condition, versus a stitch sinus. The retained sponges did not show on X-ray as it contains no radio opaque marker. Although this dose not completely abolishes the problem of missed sponges, it sure allows for intraoperative radiologic screening when the accuracy of final count is in doubt, and would have helped in clenching a correct preoperative diagnosis [6].

It is well known that surgical textile materials and instruments should be counted once at the start and twice at the conclusion of surgical procedures. However, counts are not always sufficient, since most reported cases occur in spite of a normal pack count [7]. With very few exceptions, we believe that the surgeon should not leave a sponge packed behind and proceed with other operative steps, this helps to distract his attention and the blood stained sponge will be very similar to surrounding tissues. Instead the surgeon should wait until the reason for packing e.g. oozing have stopped remove the pack and proceed with other steps. Another dangerous practice is dividing swabs into 2 or more pieces to form the so-called peanut swab this helps to mess up the final count.

## Conclusion

We recommend in addition to routine preoperative and postoperative counts, using sponges containing radio opaque markers. Sound surgical practice and removing packs as soon as their function have been achieved. Postoperative radiographic screening should be performed when counts are in doubt or high risk cases.

## Abbreviations

CT: Computed tomography
RSS: retained surgical sponge
